# Nasal Septum and External Nasal Deformity Similarities in Monozygotic Twins and Paranasal Computed Tomography Analysis

**Published:** 2018-05

**Authors:** Fatih Ozdogan, Halil Erdem Ozel, Erkan Esen, Serdar Baser, Selahattin Genc, Adin Selcuk

**Affiliations:** ENT Clinic, Derince Education and Research Hospital, Kocaeli, Turkey

**Keywords:** Nasal deformıty, Monozıgotıc twıns, Paranasal, Computed tomography analysıs

## Abstract

Since identical twins occur as a result of the division of one egg, they have the same genetic structure. Therefore, their phenotypes and sex are also always the same. However, due to the effect of environmental factors, some of the characteristics of twins living in different regions appear to develop differently. In our case of 17-year-old maternal twins, we emphasized that nasal pathologies carried a genetic background in terms of their similarity in septum deviation and external nasal deformity, which were determined to have occurred without a history of trauma.

The article has also been presented at 10^th^ Turkish Rhinology Congress, 22-25 May, 2014 Antalya, Turkey.

## INTRODUCTION

Septum deviation, the most common cause of nasal obstruction, is defined as when the septum abnormally heads toward the left or right and causes obstruction in the air passage.^1^ In a study conducted with a computed tomography (CT) scan, the incidence of septal deviation was found to be 40%.^2^ In the etiology of nasal septum deviation, genetic factors are reported to have an effect.^1^

## CASE REPORT

**Fig. 1:**
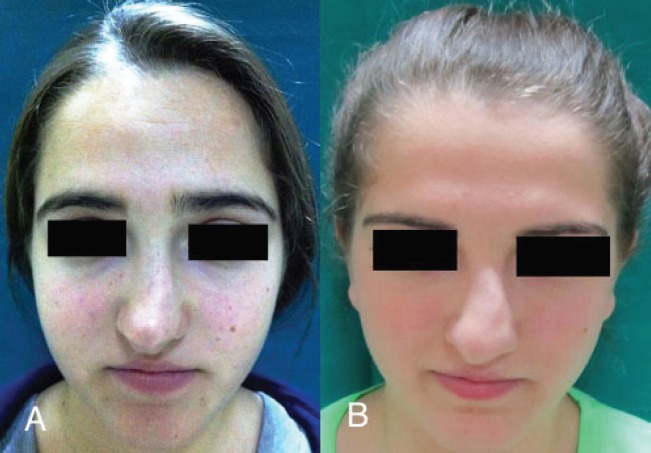
**A and B:** Preoperative frontal views.

Eighteen-year-old twin sisters were admitted to the ear-nose-throat policlinic with the complaint of an inability to breathe comfortably through their nose due to a deformity. In their medical history, the patients did not have any history of nasal trauma or surgery. Upon physical examination, the septum was observed to be deviated ahead to the left. In the right nasal passage, there was minimal crest formation and inferior turbinate hypertrophy was observed at the base. Also in the nasal dorsum, C-shaped external deformities, whose concavity was directed to the right, were observed. There were no differences in patients’ nasal examinations (Figure 1A and B). 

**Fig. 2:**
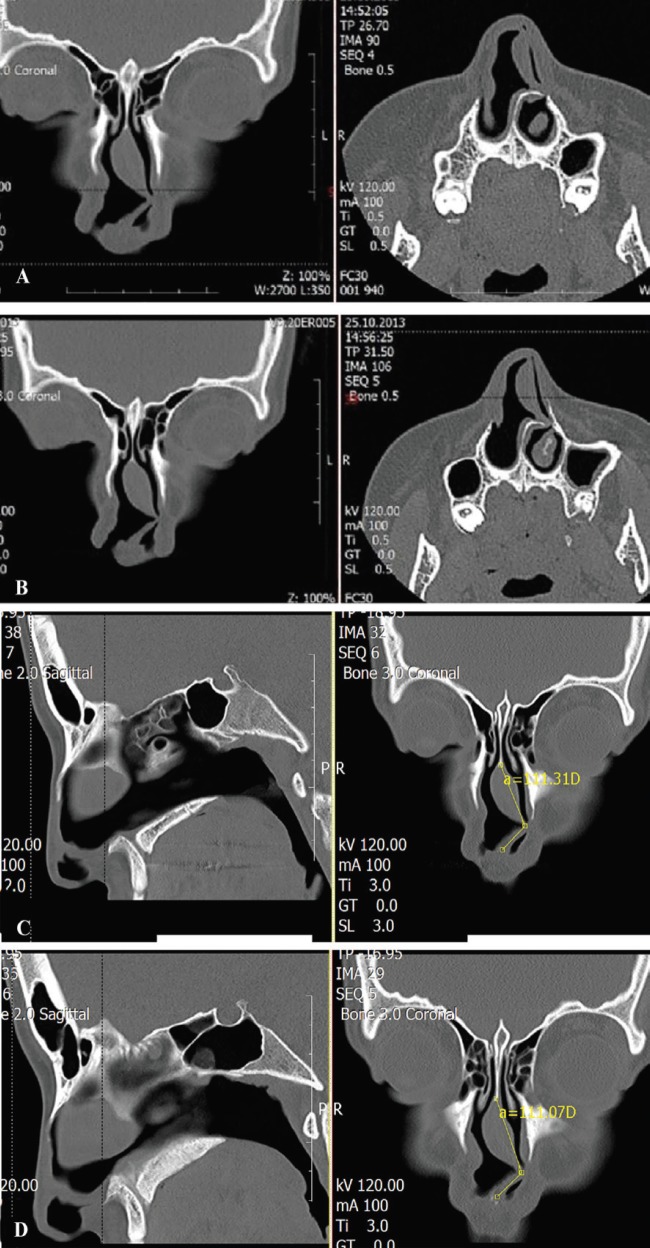
**A-D:** Preoperative paranasal sinus CT views.

**Fig. 3:**
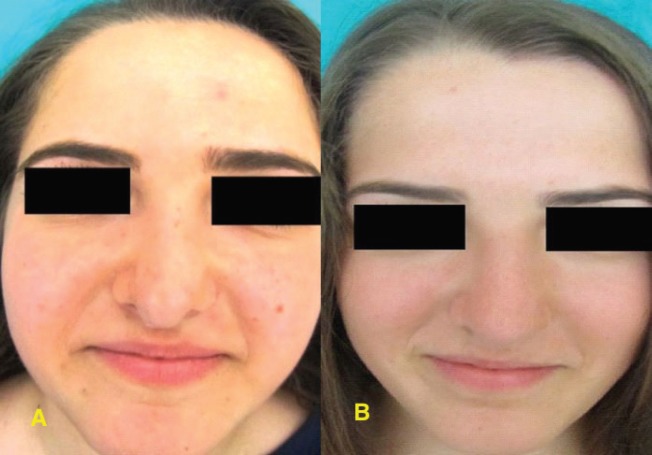
**A and B:** Nasal septal deviation angles.

In coronal sections of the patients, in the paranasal CT, it was observed that the left nasal passage was completely closed. Also, a vertical fracture line in the axial section and a spur formation in the posterior to the left were observed (Figure 2A-D). Deviated septal cartilage angles and distances were measured to be equal (nasal septal fracture angle: 111 degree) (Figure 3A and B). Open septorhinoplasty was performed via transcolumellar incision. Septoplasty, dorsal septal excision, hump excision, medial and asymmetrical lateral osteotomies were performed. Also, both patients were placed right spreader graft and nasal axis was corrected. 

## DISCUSSION

Approximately one percent of all pregnancies are twins. One-third of all twin pregnancies are seen as identical twins. In total, monozygotic twins are observed in approximately 1 in every 250 live births. Since monozygotic twins occur due to the division of a single egg, the entire genetic structure, including blood group and gender, is identical.^3^ However, due to environmental factors, some characteristics of twins living in different regions appear to develop differently. Different environmental features and different eating habits lead to the activation of different genes.^4^

In the literature related to the monozygotic twins, many genetic abnormalities were observed to be held in common: allergies, otosclerosis, hydrocephalus, hearing loss, dental defects, lip-palate defects are among the diseases. In monozygotic twins, the following examples were found in the literature associated with similarities of the nasal deformities: Howie^5^ described the genetic similarity of a nasal septum deviation in monozygotic twins, Chaiyasate *et al.*^6^ could not demonstrate a significant difference between the twins with regard to septal deformity.

In studies related to the placement of septum deviation, anterior localized deviations are emphasized in traumatic development. Posterior localized deviations are emphasized when developing in a usual genetic background.^7^ In contrast to these claims, deviation was anteriorly localized in our case. According to the Mladina classification, septal deformities are divided into seven types.^8^ Šubari´ and Mladina has found that type six septal deformities, where a horizontal groove is located in the base of anterior septal area, showed genetic transition.^9^ In our cases, deviation was anteriorly localized, but there was a vertical fracture line. 

Septal deviation is often seen with anatomic abnormalities in the neighboring region. On the concave side of septal deviation, the inferior turbinate and ethmoid bulla hypertrophy can be seen. The maxillary sinus is slightly smaller on the side of deviation. Septal deviation, especially cartilage deviation, may affect the appearance of the nasal pyramid and appear as deformities in the forms of an “S” or “C”.^10^ In our case, the C-shaped external nasal deformity was present. 

In a routine anterior rhinoscopy and radiological assessment of identical twins admitted with an inability to breathe comfortably through the nose, septal deviation and nasal deformity were demonstrated to have the same character in terms of their location and features. Absence of trauma or a history of nasal surgery in the patients emphasizes the importance of genetic maps in terms of septal deviation and nasal deformity. We suggest that our hypothesis will be stronger with additional comprehensive studies to be conducted on identical twins.

## CONFLICT OF INTEREST

The authors declare no conflict of interest.
